# Development and validation of SSR markers related to flower color based on full-length transcriptome sequencing in *Chrysanthemum*

**DOI:** 10.1038/s41598-022-26664-3

**Published:** 2022-12-24

**Authors:** Zhongya Shi, Wenqian Zhao, Zhongai Li, Dongru Kang, Penghui Ai, Hongxu Ding, Zicheng Wang

**Affiliations:** Plant Germplasm Resources and Genetic Laboratory, Kaifeng Key Laboratory of Chrysanthemum Biology, School of Life Sciences, Henan University, Jinming Road, Kaifeng, 475004 Henan China

**Keywords:** Genetic markers, Plant sciences

## Abstract

Chrysanthemum (*Chrysanthemum moriforlium* Ramat.) is one of the most popular flowers worldwide, with very high ornamental and economic values. However, the limitations of available DNA molecular markers and the lack of full genomic sequences hinder the study of genetic diversity and the molecular breeding of chrysanthemum. Here, we developed simple sequence repeat (SSR) from the full-length transcriptome sequences of chrysanthemum cultivar ‘Hechengxinghuo’. A total of 11,699 SSRs with mono-, di-, tri-, tetra-, penta- and hexanucleotide repeats were identified, of which eight out of eighteen SSR loci identified based on sixteen transcripts participated in carotenoid metabolism or anthocyanin synthesis were validated as polymorphic SSR markers. These SSRs were used to classify 117 chrysanthemum accessions with different flower colors at the DNA and cDNA levels. The results showed that four SSR markers of carotenoid metabolic pathway divided 117 chrysanthemum accessions into five groups at cDNA level and all purple chrysanthemum accessions were in the group III. Furthermore, the SSR marker CHS-3, LCYE-1 and 3MaT may be related to green color and the PSY-1b marker may be related to yellow color. Overall, our work may be provide a novel method for mining SSR markers associated with specific traits.

## Introduction

Chrysanthemum (*Chrysanthemum morifolium Ramat.*), a kind of perennial herbaceous flower of the Asteraceae family, originates from China with a cultivation history of more than 1600 years^1^. Chrysanthemum has high ornamental values due to the diverse inflorescence form and color; in addition, it also has economic values, such as edible and medicinal^1^. As a result, it has become one of the most popular and widely cultivated flowers in the world. The cultivated chrysanthemum, with a complex genetic background, is generally considered to be produced by natural hybridization of several species, including *C. lavandulifolium*, *C. indicum*, *C. zawadskii*, *C. ornatum*, *C. makinoi*, *C. japonense*, *C. vestitum*, *C. sinense*, *C. chanetii*, and *C. erubescens*^2–4^. Cultivated chrysanthemums are generally believed to be allohexaploid with a large genome size^5^. Furthermore, they possess diverse genetic variation, and up to now, there are nearly 30,000 cultivars worldwide^6^. Therefore, it is difficult to investigate the classification and genetic diversity of chrysanthemum^6,7^. Traditionally, classification, evaluation of genetic diversity, and breeding are based on morphological characteristics in chrysanthemum^8,9^. However, this method is time consuming, inefficient, and inaccurate due to environmental effects and human subjective consciousness. Molecular markers could overcome these limitations, and have been widely applied in genetic diversity assessment, population structure analysis, molecular marker-assisted selection breeding, genomic mapping, and DNA fingerprinting^10^.

Among all types of molecular markers, SSRs are one of the most crucial markers in genetic research and plant breeding, because they are codominantly inherited, abundant in the genome, highly polymorphic, and multiallelic^11,12^. SSRs, also named microsatellites, are short tandem repeat sequences of 1–6 nucleotides and distributed in the noncoding and coding regions of eukaryotic genomes^13,14^. Due to different sequence sources of SSRs, they are divided into two classes, genomic SSR (gSSR) and expressed sequence tags SSR (EST-SSR)^10^. As gSSRs are based on genomic sequences, their development is time-consuming and costly^15^, especially for non-model species with large genomes, such as chrysanthemum. EST-SSRs originate from transcriptome sequences. Therefore, they are low cost, easy to develop, and highly transferable, and may be directly related to functional genes^10,12^. In recent years, SSR markers have been reported to be widely applied in the identification and classification of cultivars, genetic diversity analysis, and molecular breeding in chrysanthemum. For example, Jo, et al. identified 16 polymorphic SSR markers based on expressed sequence tags (ESTs) in chrysanthemum^16^. These markers were then applied to classify 50 chrysanthemum cultivars and found that they were divided into four groups^16^. Luo, et al. used 10 SSR markers to classify 88 accessions of chrysanthemum and its related genera^3^. The result showed that large-flower cultivars and the wild species were divided into two clusters. Fan, et al. identified 4661 SSR loci from a chrysanthemum transcriptome and developed 361 polymorphic EST-SSR markers for further genetic mapping, molecular marker-assisted selection breeding, and genetic diversity studies in chrysanthemum^17^. However, there are still few SSR markers related to specific traits reported in the chrysanthemum.

With the development of the next-generation sequencing (NGS), it is easier, more efficient, and inexpensive to identify and develop SSR markers^10,18^. However, it is unable to obtain full-length transcripts because of the limitations of the NGS technology, such as amplification biases and short read lengths, which make it difficult to assemble the whole genome and isolate genes^19,20^. Compared with the NGS technology, single-molecule real-time (SMRT) sequencing technology, also known as the third-generation full-length transcriptome sequencing technology, has higher accuracy and longer sequencing reads^21^. Therefore, this technology has been applied to develop SSR markers in several species. For example, Wu, et al. developed the novel EST-SSRs based on full-length transcriptome data to promote conservation biology research, genetic diversity analysis, and molecular-assisted breeding in *Populus wulianensis*^10^. Xiao, et al. identified 20 polymorphic EST-SSR markers developed from sugarcane full-length expressed sequence tags and used them to perform a cluster analysis of 48 sugarcane accessions^12^. However, so far, the application of full-length transcriptome sequencing has not been reported in chrysanthemum.

Flower color is one of the most important traits for the ornamental plants, and anthocyanin and carotenoid are two major pigments of flower color^22,23^. Up to now, a set of genes involved in anthocyanin biosynthesis or carotenoid metabolism have been reported to play an important role in plant color formation. *CHALCONE SYNTHASE* (*CHS*) and *CHALCONE ISOMERASE* (*CHI*) are two key genes involved in anthocyanin biosynthesis. In *Torenia hybrida*, the flower color was modulated from blue to white through silencing of the *CHS* gene^24^. Suppression of *CHI* resulted in yellow flowers in *Dianthus caryophyllus*^25^, China aster^26^, and *Cyclamen persicum*^27^. *Malonyl-coenzyme A: anthocyanin 3-O-glucoside-6''-O- malonyl transferase* (*3MaT*) gene encodes an anthocyanin malonyltransferase, which was closely related to anthocyanin content and stability in dahlia flowers^28,29^. *LYCOPENE EPSILON CYCLASE* (*LCYE*) and *PHYTOENE SYNTHASE* (*PSY*) are two key genes involved in carotenoid metabolism^13^. In rice, the mutation of *OsLCYE* gene would increase total carotenoid content and decrease ROS accumulation under salt stress^30^. In tomato, *SlBBX20* could activate the expression of *SlPSY1*, which caused the accumulation of carotenoids and gave dark green color to the fruits and leaves^31^.

The diversity of flower color is one of the reasons why chrysanthemum has very high ornamental values. Therefore, flower color is one of the most important breeding objectives of chrysanthemum. However, few SSR markers related to flower color traits have been reported in chrysanthemum. In this study, we developed SSR markers related to flower color through full-length transcriptome sequencing in chrysanthemum. The specific objectives of our study were as follows: (1) to obtain the full-length transcriptome sequences of chrysanthemum cultivar ‘Hechengxinghuo’ and perform functional annotation; (2) to develop and validate SSR markers related to flower color based on transcripts participated in carotenoid metabolism or anthocyanin synthesis; (3) to analyze genetic diversity and classification of 117 chrysanthemum accessions with different flower colors using newly developed SSR markers. Our work not only provides new ideas for the development of SSR markers associated with specific traits, but also lays a solid foundation for the analysis of genetic diversity, classification and molecular marker-assisted breeding in chrysanthemum.

## Results

### Full-length transcriptome sequencing of chrysanthemum

Full-length transcriptome sequencing of the chrysanthemum cultivar ‘Hechengxinghuo’ was performed based on the SMRT sequencing technology. A total of 8,658,873 (7.57 Gb) clean reads were obtained with a mean clean read length (MCDL) of 1754 (Supplementary Table 2). Additionally, a total of 450,789 reads of insert (ROI) were screened from the raw sequence data, with a mean read length of insert (MRLOI) of 2359. Among the fragment sequences of inserted, the number of full-length non-chimeric reads (NFNR) was 363,653, with a full-length percentage (FLP) of 80.67%, and the average full-length non-chimeric read length (AFNRL) was 2162 (Supplementary Table 2). To determine the function of transcripts, 55,277 unigenes obtained from the transcriptome data were compared with the following protein sequence databases: KOG, KEGG^32,33^, NR, Swiss-Prot, and GO. The results showed that a total of 47,436 unigenes were annotated with an annotation rate of 85.82% (Table 1). Among all databases, the NR database had the highest annotation rate of 84.58% with 46,754 genes, while the KEGG database had 22,528 genes annotated with the lowest annotation rate of 40.75% (Table 1).

**Table 1:** **Unigene Annotation date**^32,33^**.**

Database	KOG	KEGG	NR	Swiss-Prot	GO	Total unigene	Overall annotated
Gene Number	30,158	22,528	46,754	39,356	24,341	55,277	47,436
Annotation Ratio	54.56%	40.75%	84.58%	71.20%	44.03%	–	85.82%

### Identification of the SSR locus and frequency and distribution of SSRs

**Figure 1:**
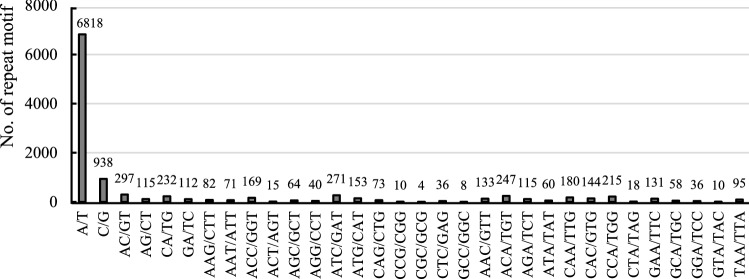
Numbers of mono-, di-, and trinucleotide repeat SSRs.

We used the MIcroSAtellite Identification Tool (MISA) to identify SSR sequences based on the transcriptome data of the chrysanthemum cultivar ‘Hechengxinghuo’. The search for perfect SSR-containing regions was restricted to motifs of mono-, di-, tri-, teta-, penta-, and hexanucleotides. The results showed that a total of 11,699 SSR motifs were identified, of which 2563 were distributed in CDS region (21.9%), 5140 in 5'-UTR (43.9%), and 3996 in 3'-UTR (34.2%) (Table 2). Additionally, 6731 mono-repeats (accounting for 86.78% of the total mononucleotides), 964 di-repeats (accounting for 88.68% of the total dinucleotide SSRs), 96 tetra-repeats (accounting for 93.20% of the total tetranucleotides) and 16 penta-repeats (accounting for 88.89% of the total pentanucleotides) occurred in UTRs (Table 2). Furthermore, 1375 tri-repeats (accounting for 51.31% of the total trinucleotides) and 31 hexa-repeats (accounting for 56.36% of the total hexanucleotides) occurred in CDS regions (Table 2). Among the SSR loci identified, mononucleotide repeats were the most abundant (7756, 66.30%), followed by trinucleotide (2680, 22.91%) and dinucleotide (1087, 9.29%) repeats, with a minority of tetranucleotide (103, 0.88%), hexanucleotide (55, 0.47%) and pentanucleotide (18, 0.15%) motif types (Table 2). The assessment of the nucleotide composition of the repeat motifs of mono-, di- and trinucleotide repeats revealed that the highest of mononucleotide repeats was A/T (6818 motifs), representing 87.91% of the total mononucleotides, the most frequent type of dinucleotide repeat was CA/TG (232 motifs), representing 21.34% of the total dinucleotides, and the most common type of trinucleotide repeat was TAC/GAT (271 motifs), representing 10.11% of the total trinucleotides (Fig. 1).

**Table 2:** Summary of Chrysanthemum SSRs identified based on the full-length transcriptome sequences.

Type	3’UTR	5’UTR	CDS	Total	Frequency (%)
Mono-	3136	3595	1025	7756	66.30
Di-	324	640	123	1087	9.29
Tri-	469	836	1375	2680	22.91
Tetra-	43	53	7	103	0.88
Penta-	8	8	2	18	0.15
Hexa-	16	8	31	55	0.47
Total	3996	5140	2563	11,699	100

### Chrysanthemum SSR primer design and marker validation

To develop SSR markers associated with flower color traits, we focused on genes participated in flower color formation and regulation. Based on the full-length transcriptome sequences of the chrysanthemum cultivar ‘Hechengxinghuo’, 16 transcripts containing SSR loci were selected. Among them, 7 transcripts were involved in carotenoid metabolism, namely IPP, PSY, PDS, ZDS, LCYE-1, LCYE-4 and NCED (Supplementary Table 3); other nine transcripts were participated in anthocyanin synthesis, namely PAL-1, PAL-2, PAL-3, CHS-1, CHS-3, CHI-1, CHI-2, DFR, and 3MaT (Supplementary Table 3). In total, 18 SSR loci were found in these transcripts. Subsequently, we designed five pair of primers for each SSR site (Supplementary Table 3) and used three chrysanthemum accessions to validate the efficacy of the newly developed SSR markers. Based on the results of polyacrylamide gel electrophoresis (PAGE), we found that 8 out of 90 SSR markers showed clear polymorphic amplified products. These markers were from 7 different transcripts, including CHS-1, CHS-3, PSY, LCYE-1, LCYE-4, CHI-1 and 3MaT (Supplementary Table 3).

### Estimation of genetic diversity using the newly developed SSR markers

**Figure 2:**
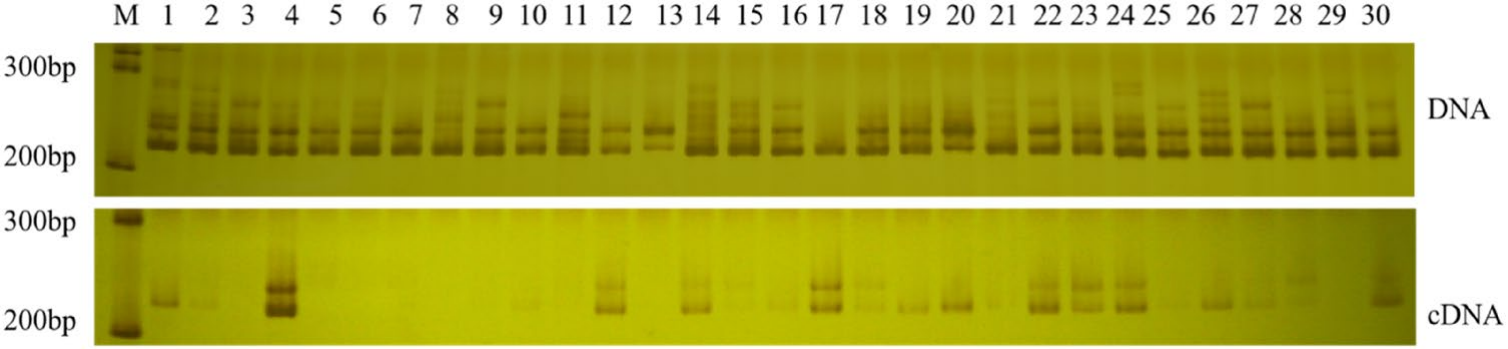
Electrophoretic map of the PSY-1a marker in some chrysanthemum accessions at the DNA and cDNA levels. Lanes 1 to 30 correspond to chrysanthemum accessions 1 to 30 in Supplementary Table 1; M: 100 bp DNA ladder marker. The images were cropped and the original images are presented in Supplementary Fig. 10.

In order to evaluate the utility of newly developed SSR markers, we used these markers to analyze the genetic diversity of 117 chrysanthemum accessions with various flower colors (Fig. 2). SSR markers can be amplified regardless of whether genes are expressed or not when the template was DNA; however, when the template was cDNA, SSR markers can only be amplified in the accessions that genes are expressed. Therefore, both DNA and cDNA of floral tissues were used as the templates to assess the genetic diversity of chrysanthemum accessions. The results showed that the number of alleles of all SSR markers was between 4 and 5 when the template was DNA, with an average of 4.5 alleles per locus, and between 2 and 8 alleles when the template was cDNA, with an average of 4 alleles per locus (Table 3). The polymorphic information content (PIC) estimated by eight markers was 0.16–0.53, with an average of 0.41 when the template was DNA, and 0.06–0.70, with an average of 0.44 when the template was cDNA (Table 3). Gene diversity (expected heterozygosity: He) ranged from 0.17 to 0.60 with an average of 0.46 when DNA was used as a template; when cDNA was used as a template, it ranged from 0.06 to 0.74 with an average of 0.49 (Table 3). The observed heterozygosity (Ho) ranged from 0.18 to 1 with an average of 0.69 when DNA was used as a template; when cDNA was used as a template, it ranged from 0 to 0.94 with an average of 0.31 (Table 3). Furthermore, the results showed that when cDNA was used as a template, the number of alleles produced by the LCYE-1 marker was the most (8), and the PIC value was 0.70 (Table 3).

**Table 3:** List of primer pairs and genetic diversity information.

Locus name	Primer sequences (5′–3′)	Repeat Motif	Distribution	Product size (bp)	*Na*		*Ho*		*He*		PIC	
					DNA	cDNA	DNA	cDNA	DNA	cDNA	DNA	cDNA
3MaT	F-TTCTTGATGGCATGGACTTC	(A)13	5′-UTR	291	5	2	0.97	0.2	0.6	0.34	0.53	0.27
	R-TTGGAAGTTGAACCAACAGC											
CHI-1	F-GCTTGCTGGTAAGTGGAAGG	(T)10	5′-UTR	219	5	4	0.85	0.33	0.59	0.39	0.53	0.36
	R-AAGCCGGCAAGTTAGTTGAT											
CHS-1	F-GCTTCAGATCTTGATATAGTTGGTG	(T)11	5′-UTR	173	4	3	0.69	0.61	0.51	0.61	0.44	0.54
	R-CGAGAATTACTCACTGCCAACA											
CHS-3	F-AATGGCGTTGAAGCTGAACT	(A)13	5′-UTR	146	4	2	0.25	0.03	0.22	0.06	0.21	0.06
	R-CGGAGGCTACAGTGGTGATT											
LCYE-1	F-AACAGTGTCCTCCTCTCCATT	(T)21	5′-UTR	204	4	8	1	0.94	0.56	0.74	0.47	0.7
	R-GGTGGCGACTGGTTATCATT											
PSY-1a	F-AACCAGTGTCCAACACAACAAC	(GT)7	5′-UTR	265	5	4	0.9	0.35	0.55	0.65	0.45	0.58
	R-ATGAGCATCACCAGCAGAGC											
PSY-1b	F-GGTGGCTAATCCAACTGGTG	(AAG)5	CDS	160	5	5	0.65	0	0.5	0.59	0.46	0.54
	R-GTTCCAGGAAGCACAATATCC											
LCYE-4	F-GCATCATAGGAGGTGTGATGG	(GAG)5	5′-UTR	185	4	4	0.18	0	0.17	0.54	0.16	0.49
	R-CGGACAATCTGGACTGAGAA											
				Average	4.5	4	0.69	0.31	0.46	0.49	0.41	0.44

*Na* Allele of number, *Ho* Observed heterozygosity, *He* Expected heterozygosity, *PIC* polymorphism information content.

### Cluster analysis of 117 chrysanthemum accessions with various flower colors

We used eight newly developed SSR markers to cluster 117 chrysanthemum accessions with diverse flower colors. Since the amplification results of SSR markers in the DNA and cDNA of the chrysanthemum flower tissues are inconsistent, the clustering analysis results are different. The results showed that the SSR marker CHS-3 or 3MaT could cluster the chrysanthemum accessions with green traits regardless of the templates being DNA or cDNA (Supplementary Fig. 2 and 4). The SSR marker CHS-1 or LCYE-4 could cluster chrysanthemum accessions with green traits when the template was DNA (Supplementary Fig. 1a and 8a), while when the template was cDNA, the clustering results of them had no correlation with flower color traits (Supplementary Fig. 1b and 8b). The CHI-1 or LCYE-1 marker could cluster the chrysanthemum accessions with green traits together when the template was DNA (Supplementary Fig. 3a and 7a), while when the template was cDNA, chrysanthemum accessions with yellow and red traits could be clustered together by CHI-1 marker and chrysanthemum accessions with green, purple and yellow traits could be clustered together by LCYE-1 marker (Supplementary Fig. 3b and 7b). When DNA was used as a template, the clustering results of the PSY-1a marker showed no correlation with chrysanthemum color traits (Supplementary Fig. 5a), but in cDNA, chrysanthemum accessions with yellow trait were clustered together (Supplementary Fig. 5b). The PSY-1b marker could cluster chrysanthemum accessions with yellow character when DNA was used as a template (Supplementary Fig. 6a), while chrysanthemum accessions with purple and yellow characters could be clustered together when the template was cDNA (Supplementary Fig. 6b). Taken together, these results implied that the markers CHS-3, 3MaT and LCYE-1 may be associated with green color and the PSY-1b marker may be associated with yellow color in chrysanthemum accessions.

The SSR markers obtained from CHS-1, CHS-3, CHI-1, and 3MaT, which are the key genes in the anthocyanin biosynthesis pathway, were integrated for cluster analysis. The results indicated that the clustering results were correlated with green traits when DNA was used as template, but these SSRs were not associated with flower color when cDNA was used as template (Supplementary Fig. 9).

**Figure 3:**
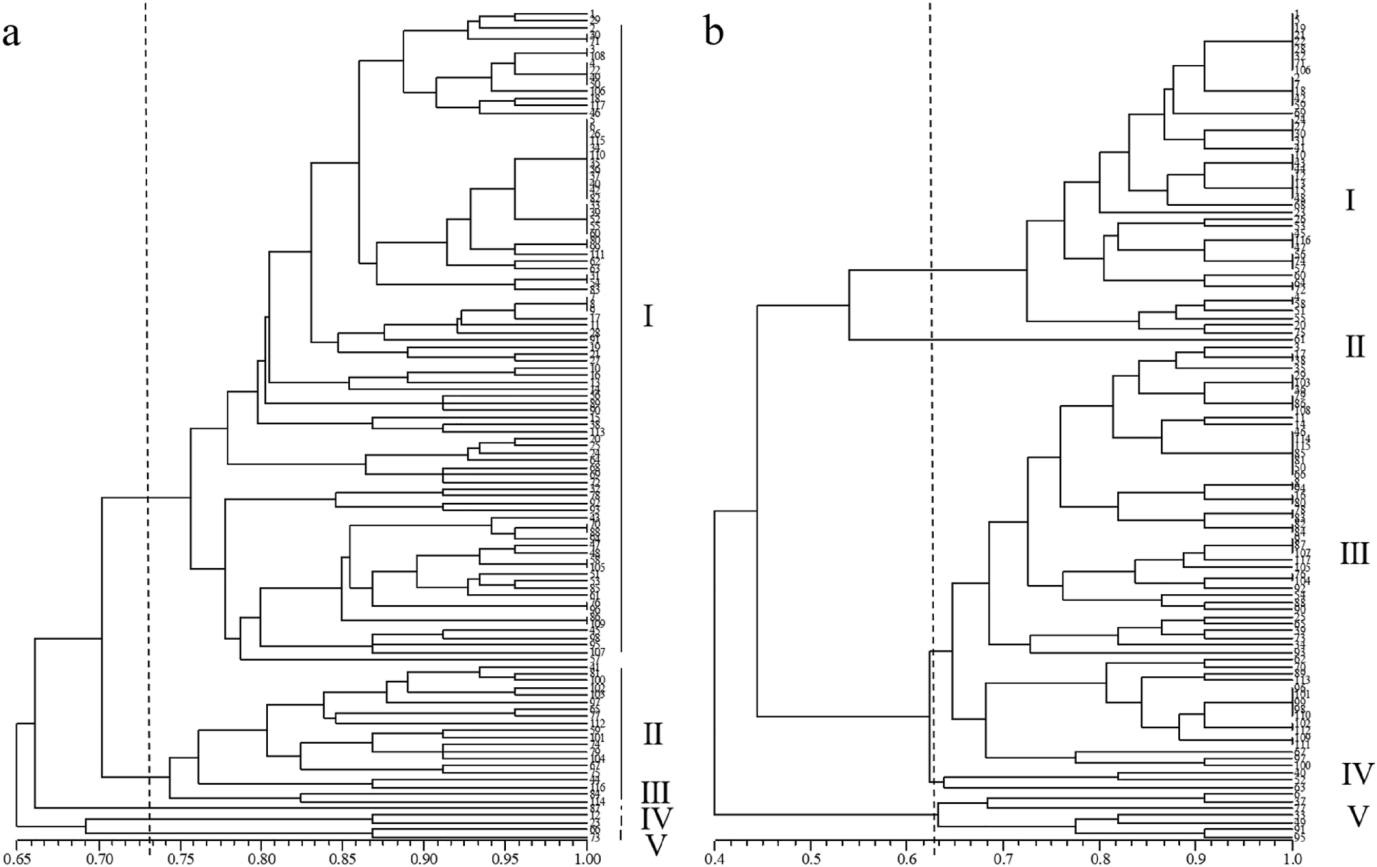
Cluster analysis of 117 chrysanthemum accessions with different colors using LCYE-1, LCYE-4, PSY-1a, and PSY-1b SSR markers at DNA (**a**) and cDNA (**b**) levels.

The SSR markers LCYE-1, LCYE-4, PSY-1a, and PSY-1b obtained from genes involved in the carotenoid metabolic pathway were used to classify 117 chrysanthemum accessions together. It was found that when DNA was used as template, 117 chrysanthemum accessions were divided into five clusters, but there was no obvious clustering in flower color (Fig. 3a). When the template was cDNA, 117 chrysanthemum accessions were divided into 5 groups at a genetic distance of 0.65 (Fig. 3b). There were 40 chrysanthemum accessions in category I (including 20 yellow, 11 red, 8 white, and 1 mixed-color chrysanthemum accessions) and 7 chrysanthemum accessions in category II (including 2 yellow, 3 red, and 2 white chrysanthemum accessions). There are 60 chrysanthemum accessions in group III (including 11 yellow, 6 red, 9 white, 10 purple, 2 green, and 22 mixed-color chrysanthemum accessions), 3 chrysanthemum accessions in category IV (including 2 red and 1 white chrysanthemum accessions), and 7 chrysanthemum accessions in category V (including 2 yellow, 2 red, 1 white, 1 green, and 1 mixed-color chrysanthemum accessions). The results showed that these markers might be related to purple, yellow and multi-color characters.

## Discussion

### Full-length transcriptome sequencing is a reliable and effective method for SSR marker development in *Chrysanthemum*

Transcriptome sequencing technology has become an efficient tool for molecular markers development due to that it can present the information of transcripts with high accuracy and wide coverage^34^. Compared to the NGS technology, the SMRT technology has the advantages of higher accuracy and longer read length^21^. Therefore, it has been widely used for the development of SSR markers in plants, especially non-model species without reference genomes. For example, Feng et al. performed the full-length transcriptome sequencing on Paulownia catalpifolia leaves treated with drought stress and identified 7367 SSRs based on the transcriptome data to promote the development of drought-resistant SSR markers^35^. Xiong et al. developed 49 polymorphic SSR markers within the full-length transcriptome sequences of Kengyilia melanthera and used them to analyze the genetic relationships of 56 K. melanthera accessions^34^. In this study, the third-generation transcriptome sequencing was performed to obtain the full-length transcriptome sequences in chrysanthemum cultivar ‘Hechengxinghuo’. A total of 8,658,873 (7.57 Gb) clean reads were obtained including 363,653 full-length non-chimeric reads, which was similar with the number of that in *P. catalpifolia*^35^ (349,745) but less than *K. melanthera*^34^ (491,001). In addition, a total of 55,277 unigenes were identified with the average length of 2385 bp in chrysanthemum cultivar ‘Hechengxinghuo’, while the mean unigene length was 585 bp in *Chrysanthemum nankingense*^14^, 727 bp in diploid *Chrysanthemum indicum*^36^, and 784 bp in tetraploid *C. indicum*^36^, each of which were sequenced using the NGS technology. These results indicated that the transcripts derived from the SMRT technology were longer in length than those derived from the NGS technology. In our work, a total of 47,436 unigenes were successfully annotated with the annotation ratio of 85.82%, which was higher than the annotation ratio of diploid *C. indicum* (74.60%)and tetraploid *C. indicum* (73.60%) obtained using the NGS technology^36^. These may be due to the long read length and high accuracy of the SMRT technology^37^. Based on the full-length transcriptome sequences, we identified 11,699 SSR loci and developed eight polymorphic SSR markers related to flower color, and the newly developed SSRs were used for genetic diversity analysis and classification of chrysanthemum accessions. To the best of our knowledge, this is the first study to develop SSR markers using the SMRT technology in *Chrysanthemum*. Our results demonstrated that full-length transcriptome sequencing is a reliable and effective method for SSR marker development in *Chrysanthemum*.

### Distribution and frequency of SSRs in transcriptome

SSR molecular markers have been found to have non-random distribution in gene regions, including CDS and UTRs^38^. The results of our study showed that most SSRs were distributed in the 5'UTR region (43.9%), followed by the 3'UTR (34.2%) and CDS regions (21.9%) (Table 2), which was similar with other species^34^. The possible reason is that the mutation of SSRs will result in severe change of the structure and function of genes when SSRs locate in CDS regions^39^. In addition, we found that most of mono- (86.78%), di- (88.68%), tetra- (93.20%), and pentanucleotide (88.89%) repeats located in UTRs, while over half of trinucleotide (51.31%) and hexanucleotide (56.36%) repeats located in CDS regions (Table 2). The similar results were also detected in many other species, such as coconut^40^, eggplant^41^, and castor bean^42^. The reason may be that trinucleotide and hexanucleotide repeats are less likely to cause frameshift mutations^40,42^. In terms of the SSR repeats, the most frequent repeat type was mononucleotide (66.30%), followed by trinucleotide (22.91%) and dinucleotide (9.29%) repeats, which was similar with the result in Populus wulianensis^10^. However, the most abundant repeat motif was trinucleotide in coconut^40^, eggplant^41^, castor bean^42^, and Sugarcane^12^. Additionally, we found A/T was the dominant mononucleotide repeat in chrysanthemum, which was consistent with the eukaryotes^43^. However, the AC/GT repeats were the most abundant dinucleotide motifs in our work, which was different from most other plants in which the AG/CT repeats were the most common dinucleotide repeats^40^. These differences in frequency of SSR repeats may be attributed to the differences in species, development tools, or SSR searching criteria^10^.

### Development and application of SSR markers associated with flower color

SSRs, as one of the most valuable molecular markers, have been widely applied in identification and classification of cultivars^44^, assessment of genetic diversity^45,46^, exploration of genetic relationship and intraspecific genetic divergence^3,36^. However, few reports are available on the development of trait-associated SSR markers. Xia et al. developed 191 polymorphic SSR markers based on the transcriptome sequences and found nine SSR markers significantly associated with plant height through association analysis in coconut^40^. In this study, we developed eight polymorphic SSR markers associated with flower color based on seven transcripts (Table 3), which derived from five genes involved in carotenoid metabolism or anthocyanin synthesis, including *CHS*, *CHI*, *3MaT*, *LCYE* and *PSY*. Among them, *CHS* and *CHI* are two key genes in the anthocyanin biosynthetic pathway. The pigment content of radish fleshy root was highly correlated with the expression level of *CHS* gene^47^, and *CHI* played an essential role in seed, fruit, and flower color formation^22,44^. 3MaT, encoding an anthocyanin malonyltransferase, has been reported to be closely related to the content and stability of anthocyanin in dahlia flowers^28,29^. Additionally, *LCYE* and *PSY* are two key genes in the carotenoid metabolic pathway^13^. The mutant of *OsLCYE* gene had higher carotenoid content than wild-type plants under salt stress^30^. PSY catalyzes the first step of the carotenoid biosynthetic pathway and is an important rate-limiting enzyme of carotenoid biosynthesis^48^. Furthermore, it has been reported that anthocyanin and carotenoids are main pigments of flower color^22,23^. Therefore, the eight newly developed SSR markers in this study are likely to be related to flower color.

The PIC values represent the informativeness of molecular markers, which was categorized as low (PIC < 0.25), moderate (0.5 < PIC < 0.25), and high (PIC > 0.5), respectively^49^. In this study, we used eight newly developed SSR markers to evaluate the genetic diversity of 117 chrysanthemum accessions at DNA and cDNA levels. These SSRs exhibited moderate PIC values ranged from 0.16 to 0.53 with an average of 0.41 and from 0.06 to 0.70 with an average of 0.44 at DNA and cDNA levels (Table 3), respectively, which were lower than that of chrysanthemum cultivars reported previously by Jo, et al. (0.15–0.89, 0.57)^16^, Luo, et al. (0.52–0.94, 0.79)^3^, and Fan et al. (0.29–0.86, 0.67)^17^, but higher than *K. melanthera* (0.025–0.431, 0.240) and *Phaseolus vulgaris* (0.12–0.85, 0.47)^50^. In addition, we found the PIC value at cDNA level was higher than that of DNA level (Table 3), implying that the SSRs presented higher levels of informativeness at the transcriptional level. In addition, These results indicated that the newly developed SSR markers in our work had the potential for further genetic study in chrysanthemum and its relatives.

In recent years, SSR markers have been reported to be widely used for classification in Chrysanthemum. Zhang et al. used SSR molecular markers to identify and classify 480 Chinese traditional ornamental chrysanthemum cultivars^44^. Feng et al. used SSR markers to analyze the phylogenetic relationship of 32 medicinal chrysanthemum cultivars and found they were divided into two group and group I included all the “Machengju” and “Hangju” samples^45^. Olejnik et al. used 14 polymorphic SSRs to classify 97 chrysanthemum cultivars^46^. The results showed that all the cultivars were divided into four clusters and the first cluster contained only small-flowered accessions. In this study, we used the eight newly developed SSR markers to cluster 117 chrysanthemum accessions with diverse flower colors. We found that four SSR markers, LCYE-1, LCYE-4, PSY-1a, and PSY-1b, divided 117 chrysanthemum accessions into five groups at DNA and cDNA level, but there was no obvious clustering of flower color in chrysanthemums at the DNA level. At cDNA level, all purple chrysanthemum accessions were in the group III (Fig. 3b), implying that these four SSR markers may be correlated with purple color. Furthermore, the results of cluster analysis implied CHS-3, 3MaT and LCYE-1 markers may be related to green color and PSY-1b marker may be related to yellow color (Supplementary Fig. 2, 4, 6 and 7).

Taken together, our work is a new attempt to develop SSR molecular markers related to specific trait based on the full-length transcriptome sequences, and will lay a solid foundation for genetic diversity analysis, classification and molecular-assisted breeding in *Chrysanthemum*.

## Materials and methods

### Plant materials

The plant experiments were performed in accordance with relevant guidelines and regulations. The chrysanthemum cultivar ‘Hechengxinghuo’ used for full-length transcriptome sequencing is maintained by the Key Laboratory of Chrysanthemum Biology of Kaifeng City, Henan University, China. The 117 chrysanthemum accessions used to validate the newly developed SSR markers were preserved in the Longting Park (Kaifeng, Henan Province, China), and the flower color of chrysanthemum accessions was recorded with the RHS Mini Color Chart (Supplementary Table 1).

### RNA extraction, SMRTbell library preparation, and transcriptome sequencing

Total RNA was extracted from the flowers of the chrysanthemum cultivar ‘Hechengxinghuo’ using the RNAprep Pure Plant Plus Kit (Tiangen, Beijing, China) following the manufacturer’s protocol. The integrity of the RNA was then tested using an Agilent 2100 instrument. The cDNA was synthesized using a SMARTer® PCR cDNA Synthesis Kit (Roche, Switzerland). To construct the SMRTbell library, PCR amplification was performed with KAPA HiFi PCR Kits (Roche, Switzerland) and the amplified products were used to generate the SMRTbell library with the SMRTbell template prep kit 1.0. After library construction, a certain concentration and volume of library template and enzyme complex were transferred to the nanopore of PacBio Sequel sequencer to start real-time single-molecule sequencing (Nextomics Biosciences, Wuhan, China).

### Analysis of transcriptome data and microsatellite mining

After sequencing, high-quality transcriptome data was obtained by filtering and the clean data was processed using PacBio SMRT Link version 5.1. To obtain annotation information of the transcripts, the non-redundant transcript sequences obtained were aligned to the NR, SwissProt, GO, COG, KOG and KEGG databases using BLAST software (version 2.2.26)31. The full-length consensus sequence was used for subsequent analysis. Potential SSRs included in transcript sequences were searched and analyzed using MISA32 with default parameters. The acquired SSRs were containing basic motifs with mono-, di-, tri-, tetra-, penta-, and hexanucleotide repeats.

### Chrysanthemum SSR primer design

Transcripts related to flower color formation and regulation were selected to develop SSR markers. Oligonucleotide primers were designed according to the flanking sequences of the SSRs using the Primer 3.0 software. Potential SSR markers were selected according to the following parameters: primer length between 18 and 22 bp, PCR product length between 100–300 bp, primer melting temperature (Tm) between 55 and 65 °C and GC content of 40–60%.

### DNA and RNA extraction from floral materials of 117 chrysanthemum accessions

Total DNA was extracted using the Plant Genomic DNA Extraction Kit (Sangon Biotech, Shanghai, China) and the RNA was extracted using the Spin Column Plant Total RNA Purification Kit (Sangon Biotech) following the manufacturer's protocol. The RNA (1.0 μg) was reverse-transcribed into cDNA using the PrimeScript RT reagent Kit (Takara Bio, Tokyo, Japan) following the manufacturer's protocol. The DNA and cDNA concentration of each sample was tested using a NanoDrop2000 spec-trophotometer (Thermo Fisher Scientific, USA) and adjusted to 20 ng/μl in the final.

### PCR validation of SSR markers

PCR was performed to validate the SSR markers. Each 20 μl PCR reaction contained 10 μl PCR Mix, 1 μl 10 mM forward primer, 1 μl 10 mM reverse primer, 1 μl 20 ng/μl DNA template, and 7 μl H2O. PCR conditions were used as follows: 95 °C for 5 min; 32 cycles of 95 °C for 30 s, 55 °C for 30 s and 72 °C for 30 s; and a final step at 72 °C for 10 min. The temperature was then held at 12 °C. The PCR products were first detected by 1% agarose gel electrophoresis, then detected by 8% non-denaturing polyacrylamide gel electrophoresis, and then photographed after silver staining.

### Analysis of genetic diversity and classification

The genetic diversity of the 117 chrysanthemum accessions was examined using the newly developed SSR markers. The individual band amplified by the SSR primers in the SSR banding profile was scored as present (1) or absent (0). The polymorphic information content (PIC), the number of alleles (Na), and gene diversity were calculated by the PowerMarker 3.25 software. The observed heterozygosity (Ho) and expected heterozygosity (He) were determined by Popgen32. Cluster analysis of the SAHN model using the UPGMA method was performed using NTSYS-pc version 2.1.

## Supplementary Information

Supplementary Information 1.

Supplementary Information 2.

## Data Availability

The raw sequence of the full-length transcriptome used in this study is available at the National Center for Biotechnology Information (NCBI) under BioProject ID is PRJNA888146 and the URL is https://www.ncbi.nlm.nih.gov/bioproject/?term=PRJNA888146.
