# Supplementary material for: Development and validation of SSR markers related to flower color based on full-length transcriptome sequencing in Chrysanthemum
Source: Sci Rep. 2022 Dec 24;12:22310. doi: 10.1038/s41598-022-26664-3 (PMC9789954; doi:10.1038/s41598-022-26664-3)
Supplement: Supplementary file 1 — Supplementary Information 1. [file 41598_2022_26664_MOESM1_ESM.pdf]

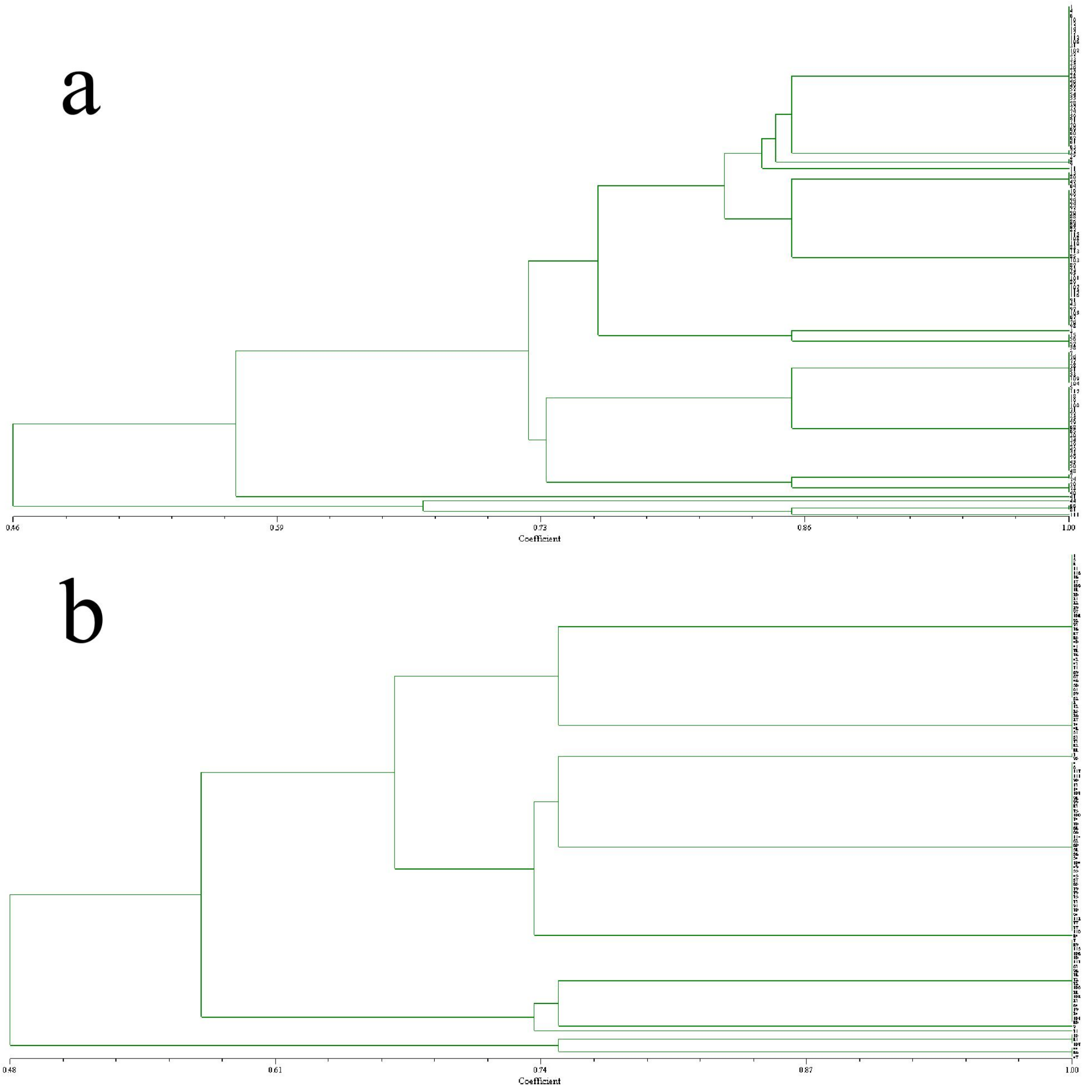

Supplementary Fig 1 Clustering analysis of 117 chrysanthemum accessions with various flower colors by CHS-1 SSR marker.

a. Results of clustering analysis with DNA as template; b. Results of clustering analysis with cDNA as template.

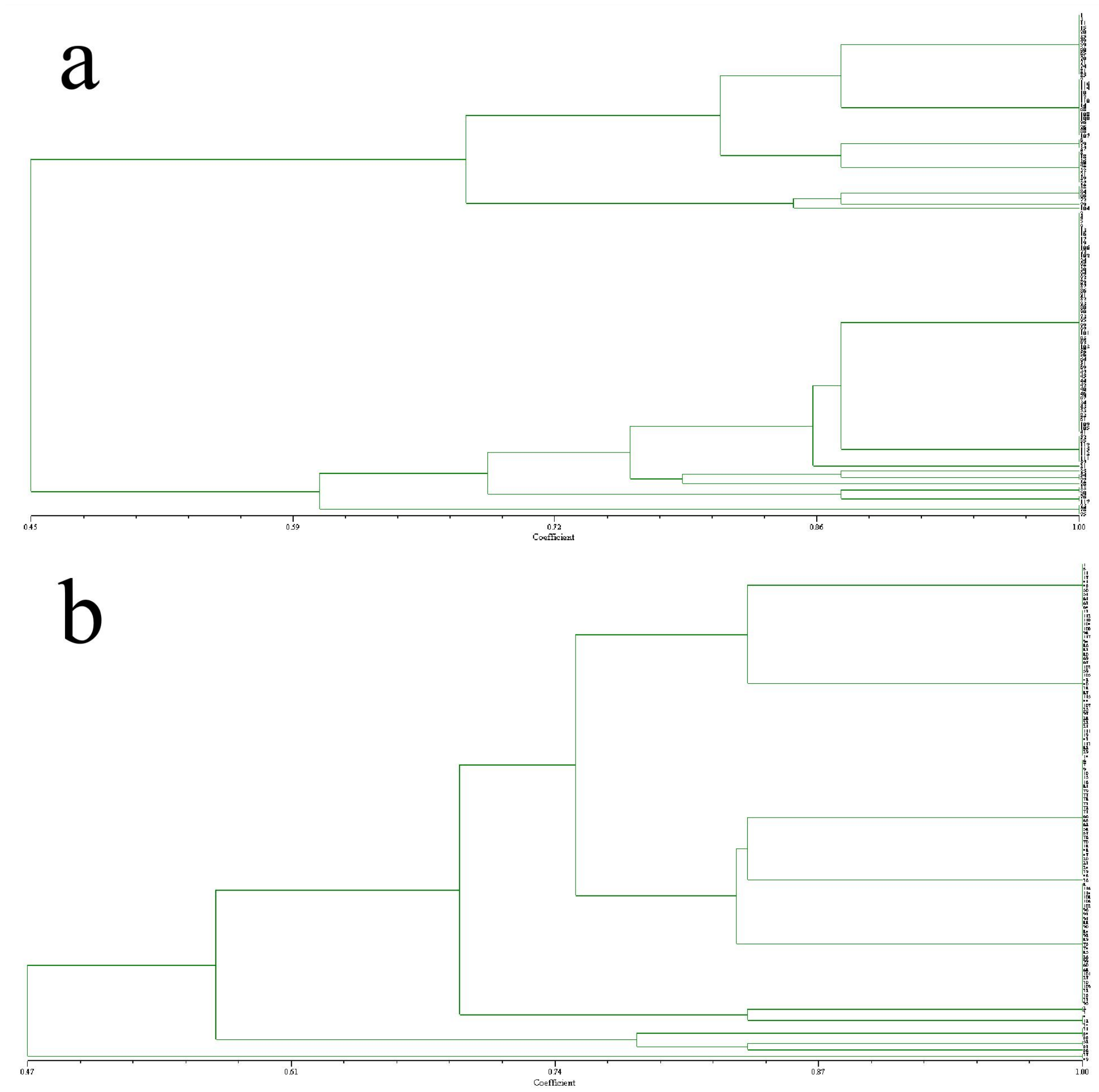

Supplementary Fig 2 Clustering analysis of 117 chrysanthemum accessions with various flower colors by CHS-3 SSR marker.

a. Results of clustering analysis with DNA as template; b. Results of clustering analysis with cDNA as template.

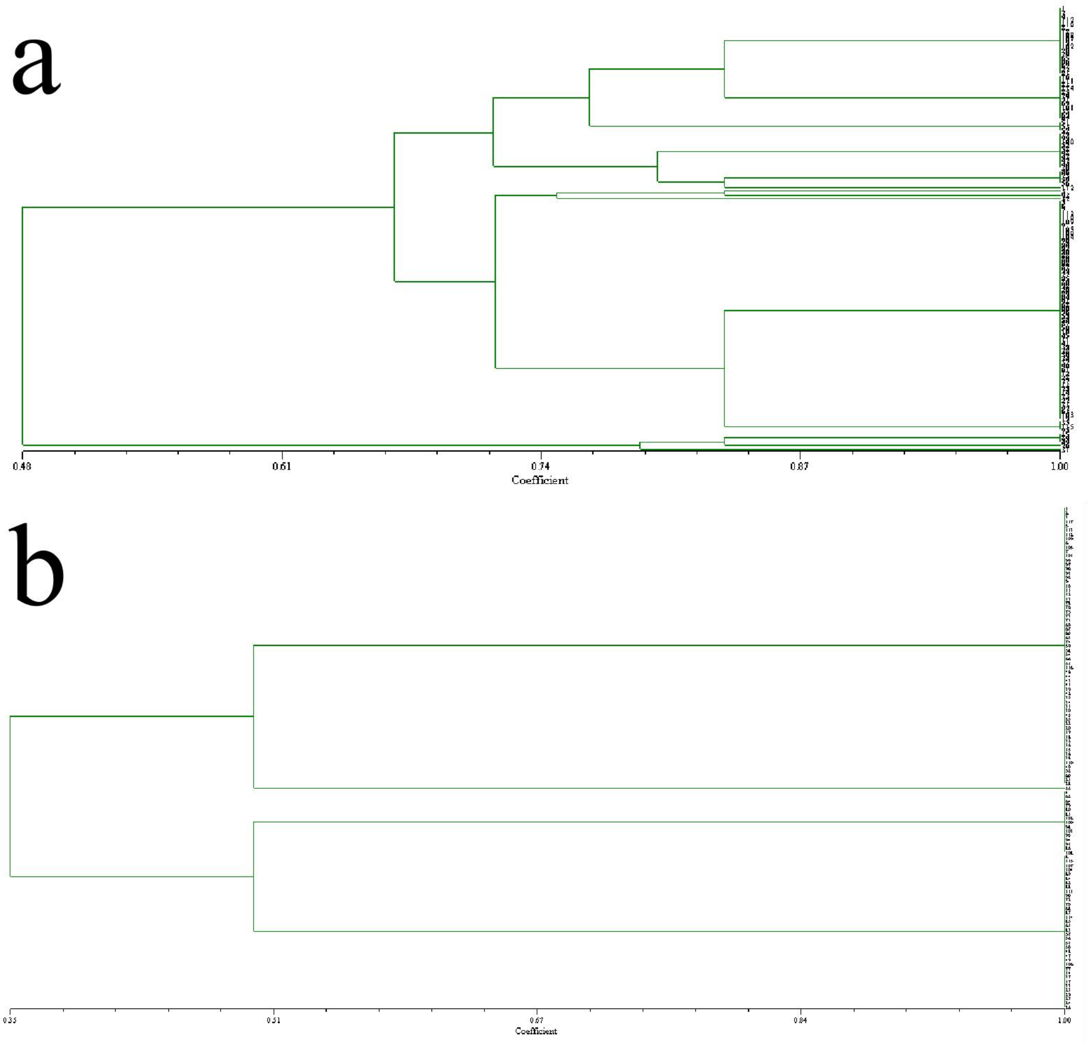

Supplementary Fig 3 Clustering analysis of 117 chrysanthemum accessions with various flower colors by CHI-1 SSR marker.

a. Results of clustering analysis with DNA as template; b. Results of clustering analysis with cDNA as template.

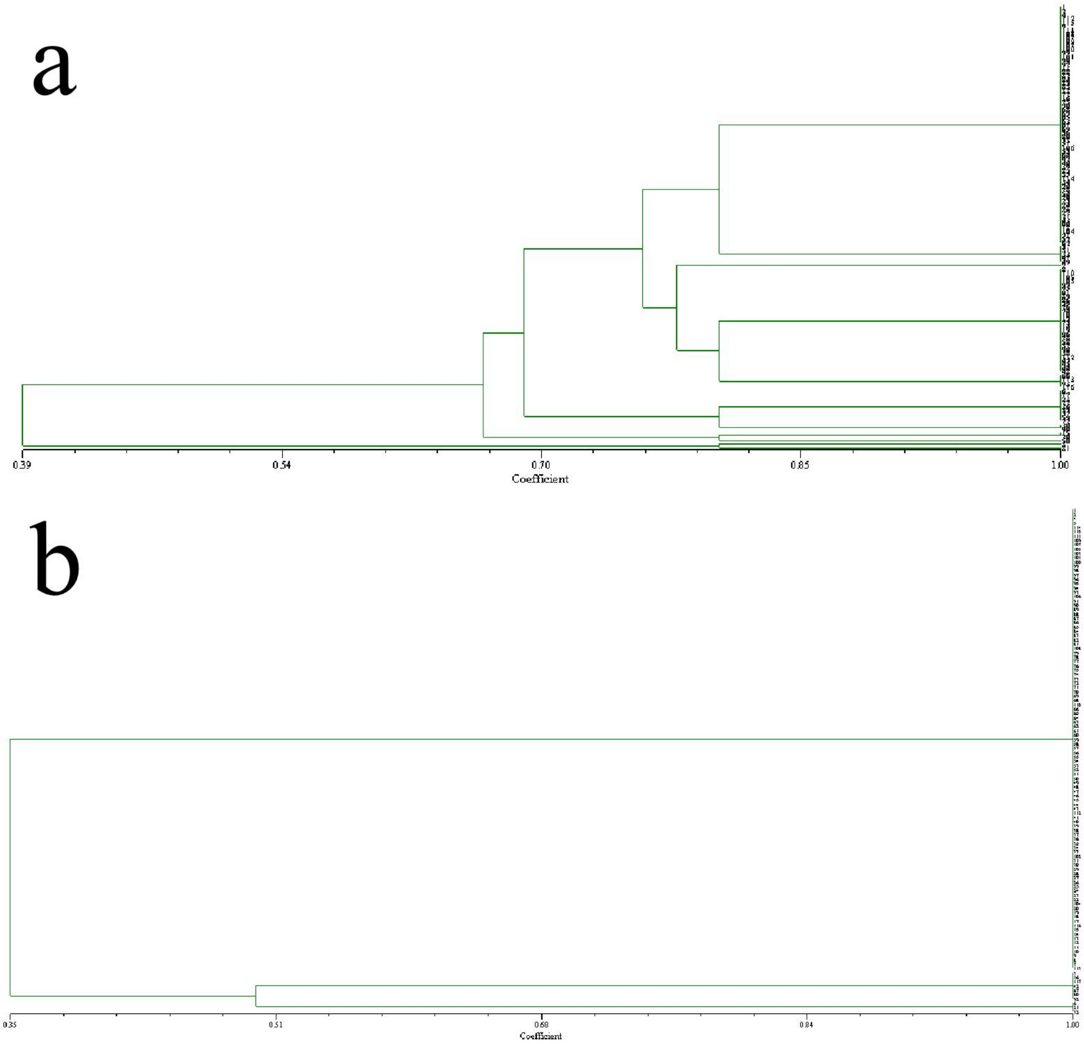

Supplementary Fig 4 Clustering analysis of 117 chrysanthemum accessions with various flower colors by 3MaT SSR marker.

a. Results of clustering analysis with DNA as template; b. Results of clustering analysis with cDNA as template.

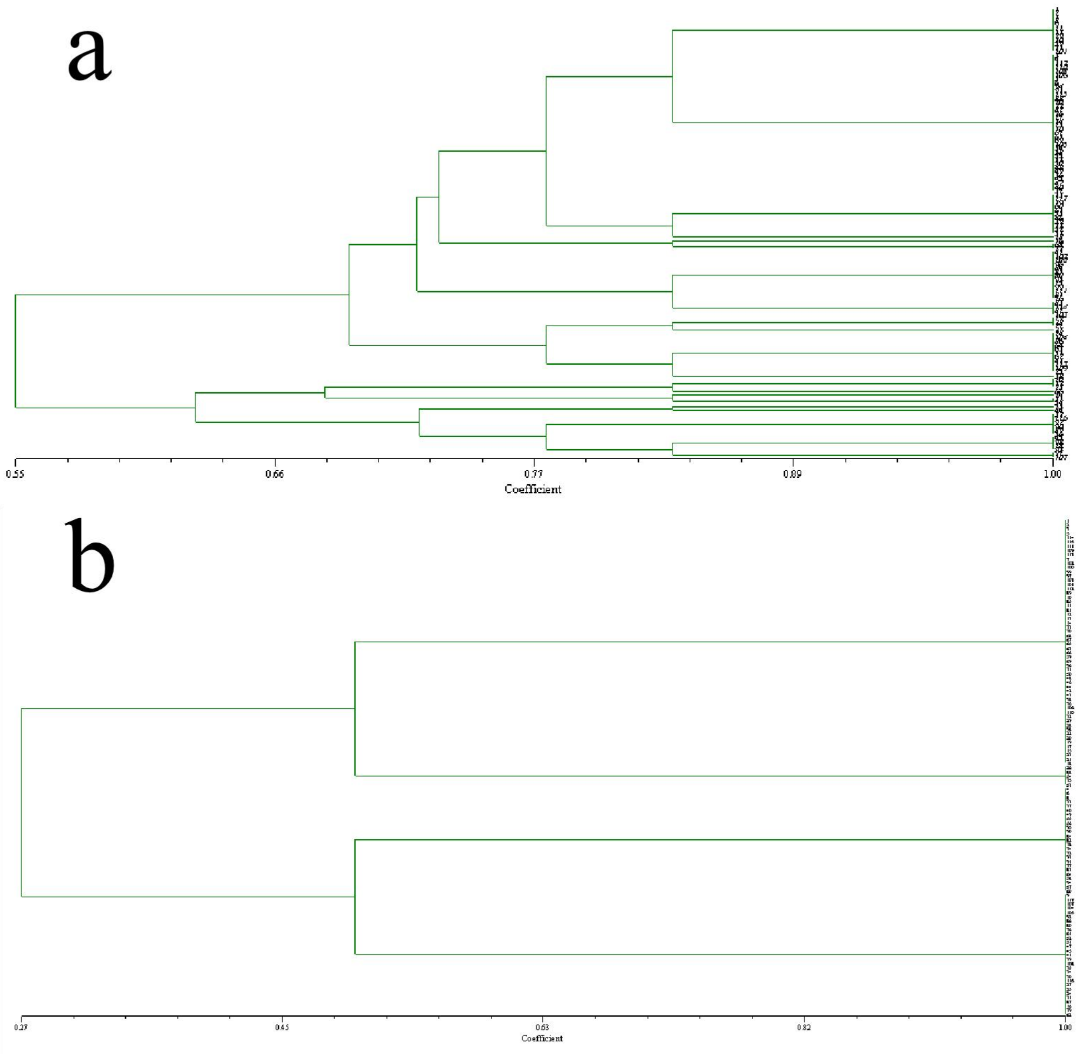

Supplementary Fig 5 Clustering analysis of 117 chrysanthemum accessions with various flower colors by PSY-1 SSR marker.

a. Results of clustering analysis with DNA as template; b. Results of clustering analysis with cDNA as template.

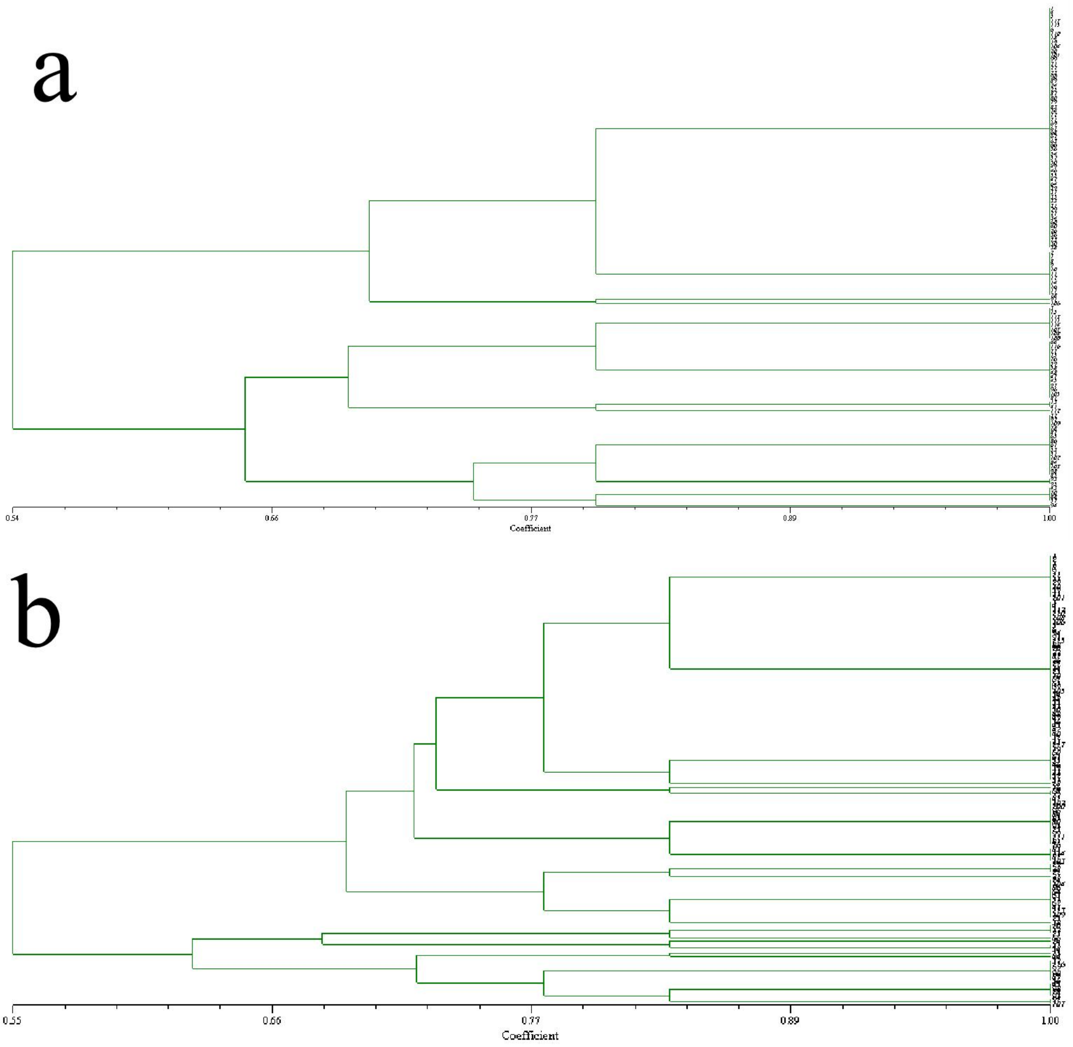

Supplementary Fig 6 Clustering analysis of 117 chrysanthemum accessions with various flower colors by PSY-2 SSR marker.

a. Results of clustering analysis with DNA as template; b. Results of clustering analysis with cDNA as template.

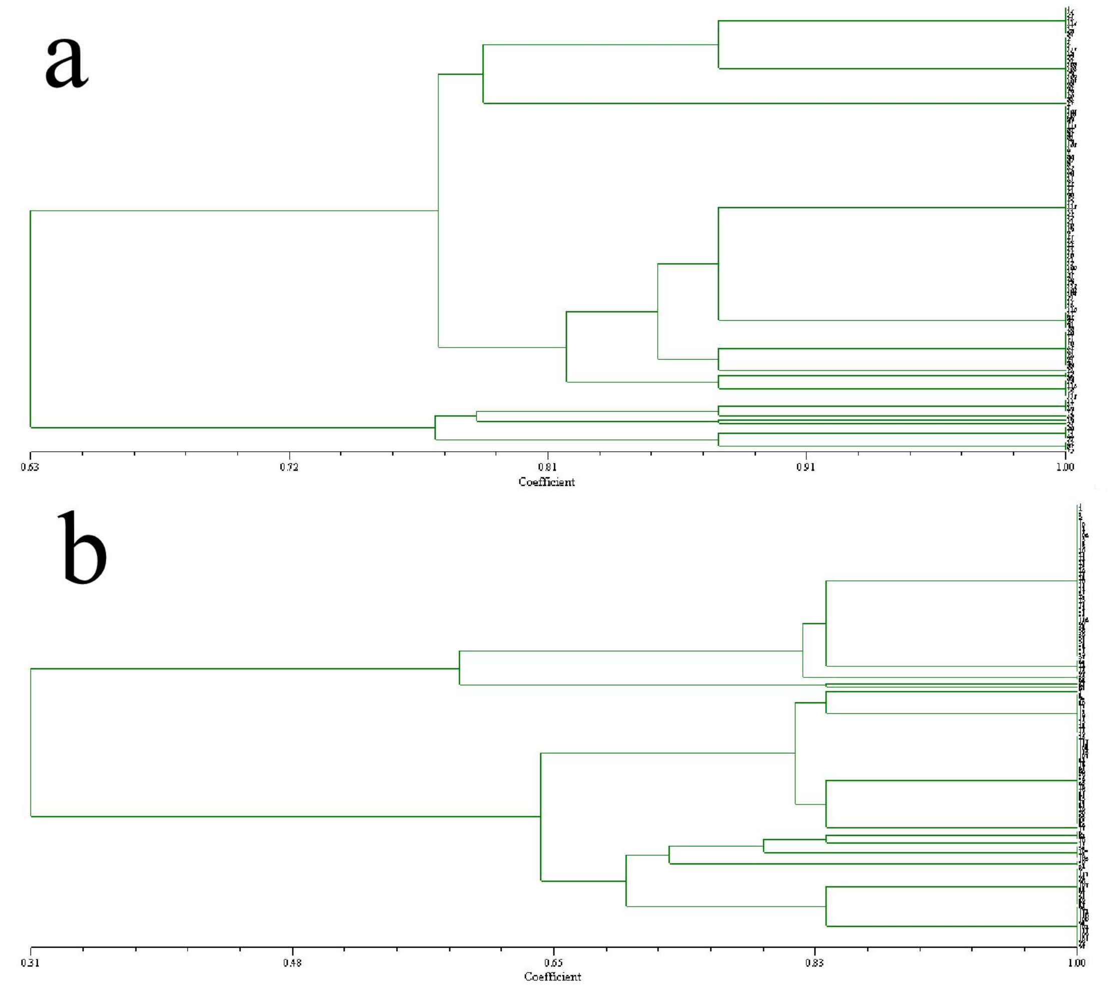

Supplementary Fig 7 Clustering analysis of 117 chrysanthemum accessions with various flower colors by LCYE-1 SSR marker.

a. Results of clustering analysis with DNA as template; b. Results of clustering analysis with cDNA as template.

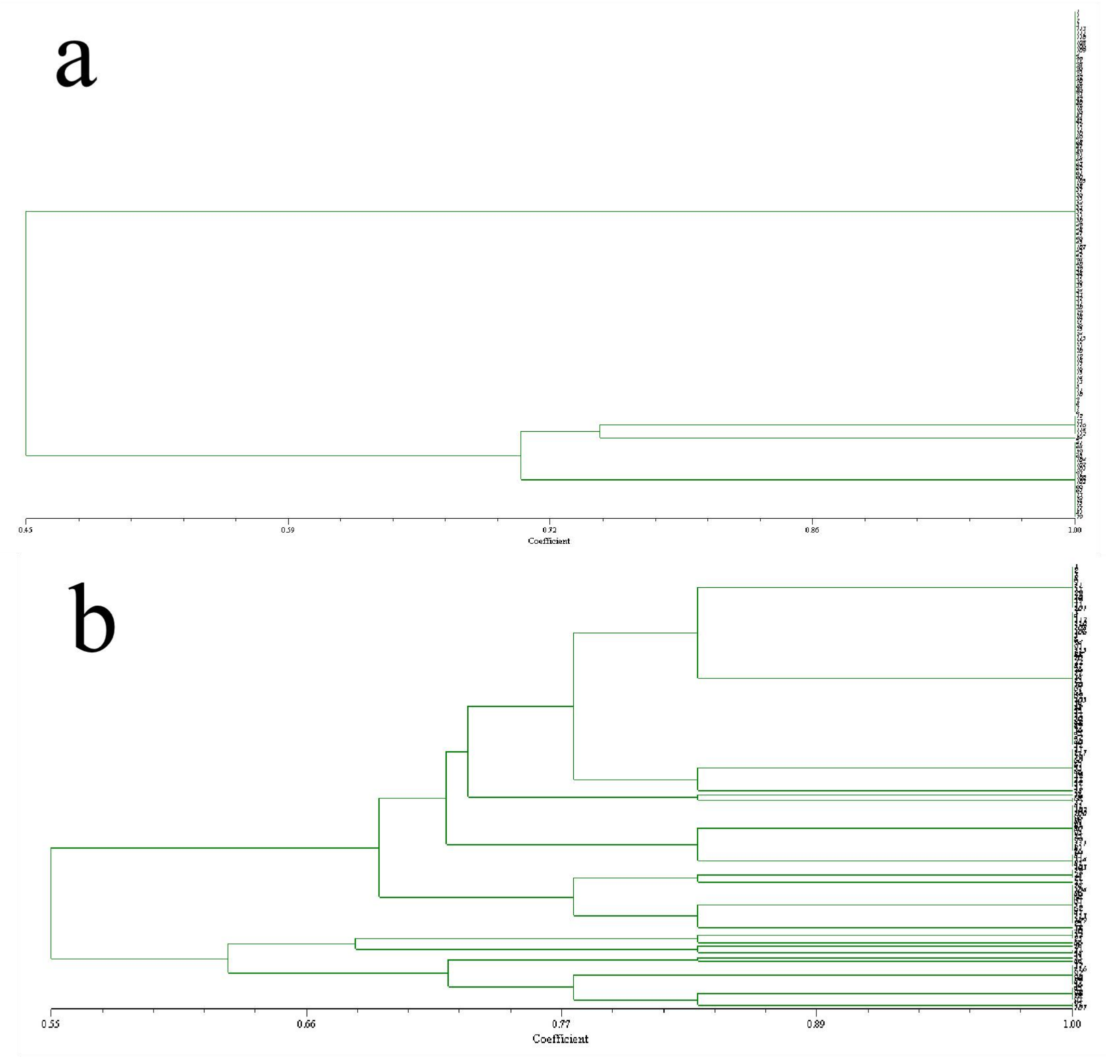

Supplementary Fig 8 Clustering analysis of 117 chrysanthemum accessions with various flower colors by LCYE-4 SSR marker.

a. Results of clustering analysis with DNA as template; b. Results of clustering analysis with cDNA as template.

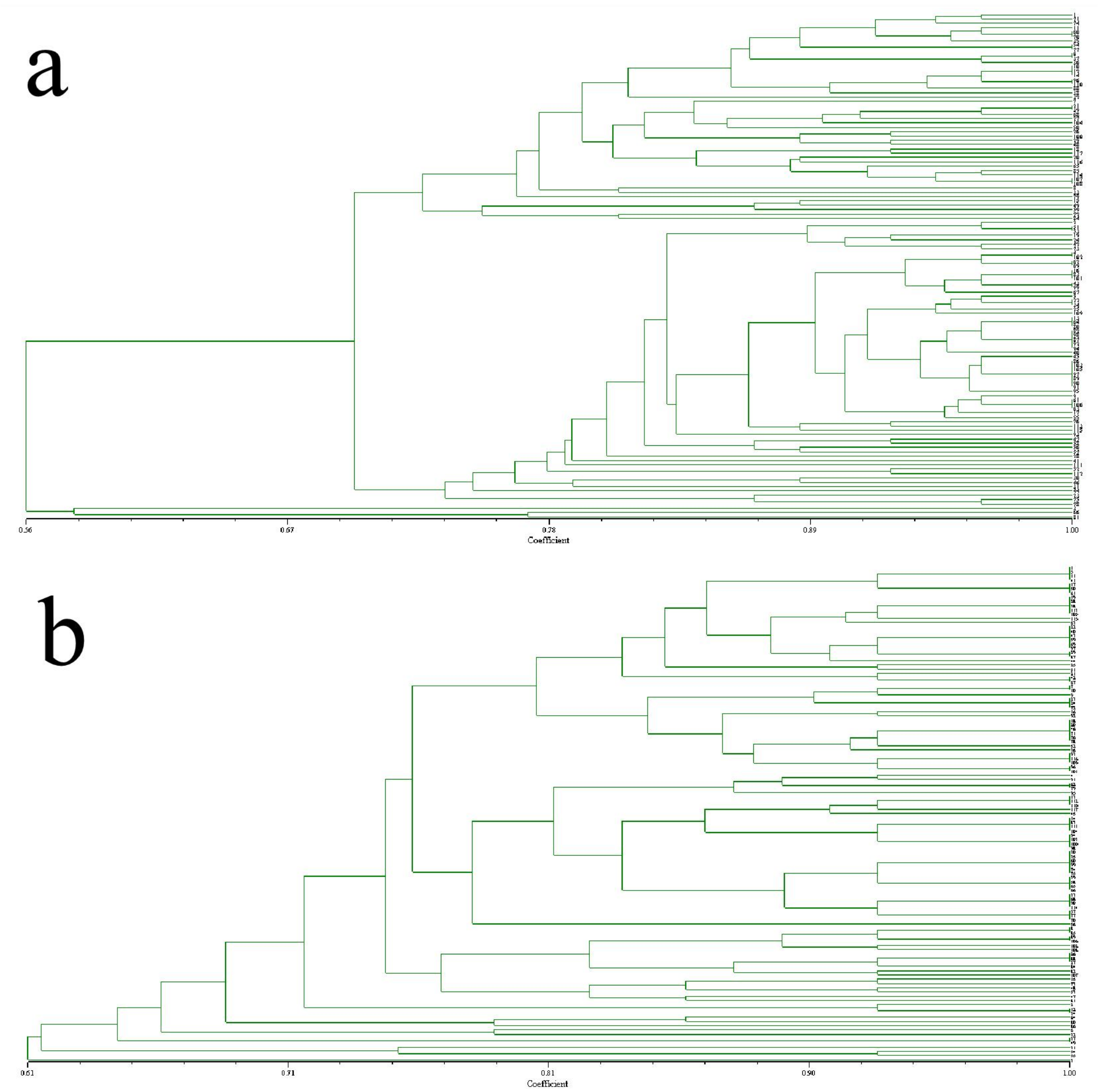

Supplementary Fig. 9 Clustering analysis of 117 chrysanthemum accessions with various flower colors by SSR markers involved in the anthocyanin biosynthesis pathway.

a. Results of clustering analysis with DNA as template; b. Results of clustering analysis with cDNA as template.

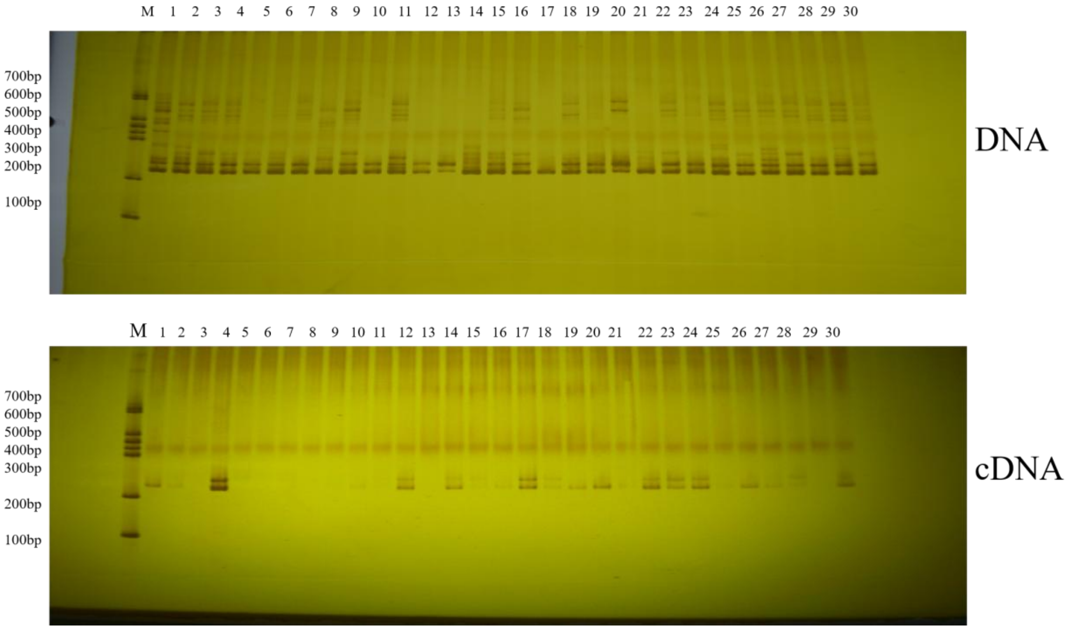

Supplementary Fig. 10 Electrophoretic map of the PSY-1a marker in some chrysanthemum accessions at the DNA and cDNA levels.

Supplementary Table 1 117 chrysanthemum accessions with different colors.

No	Variety	Color number	Flower image
1	Guohuahuangyueshan	RHS6A	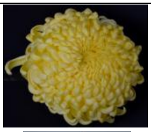
2	Shenguanghuabao	RHS22A	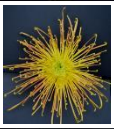
3	Xuelingqingyun	RHS4D	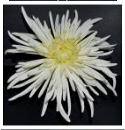
4	Yegonghaolong	RHS6A	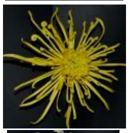
5	Gushuiliuxia	RHS4A	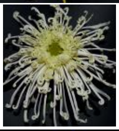
6	Huangshiba	RHS6A	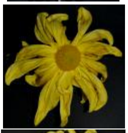
7	Guohuajindashe	RHS4A	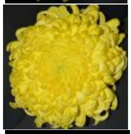
8	Dulijinqiu	RHS6A	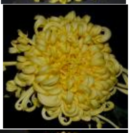
9	Yanshanjinshi	RHS6A	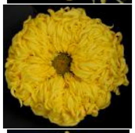
10	Panlongjinzhuo	RHS7D	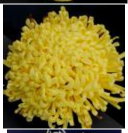
11	Tangyutianjv	RHS8C	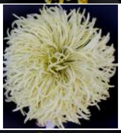
12	Panlongjinzhuo	RHS4A	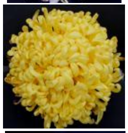
13	Longyunxingkong	RHS8C	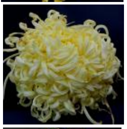
14	Jinfoxiufa	RHS12A	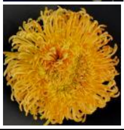

15	Baixuegongzhu	RHS4D	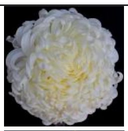
16	Jinkuixiangyang	RHS4A	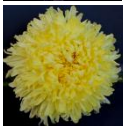
17	Jinfenghuanchao	RHS6A	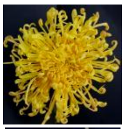
18	Xingshitu	RHS8C	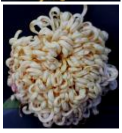
19	Shengnongzhuang	RHS39B	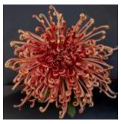
20	Guohuajinhua	RHS6A	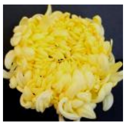
21	Yuezhiguang	RHS4A	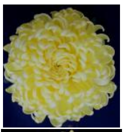
22	Guohuayueshan	RHS7D	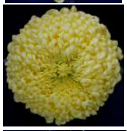
23	Hongshixingmeng	RHS8C	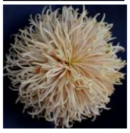
24	Fenglinfengwu	RHS7D	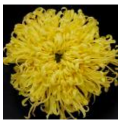
25	Ruyijingou	RHS6A	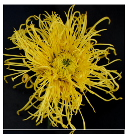
26	Huangxixiang	RHS8C	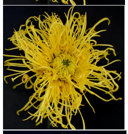
27	Jinefeitian	RHS4A	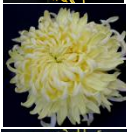
28	Jinxiaguan	RHS6A	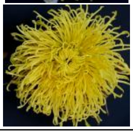

29	Guohuayueshan	RHS8C	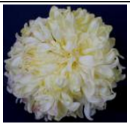
30	Quanxiangbaxi	RHS15B	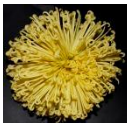
31	Gangtiecaixian	RHS12A	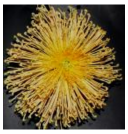
32	Panlongjinzhong	RHS6A	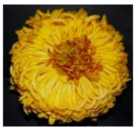
33	Quanxiangshuibajiao	RHS7D	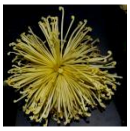
34	Panlongjinwei	RHS4A	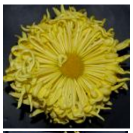
35	Shanyangchisong	RHS14A	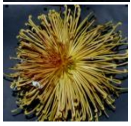
36	Fenshiba	RHS70B	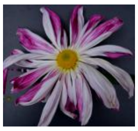
37	Huanshuiheixuanfeng	RHS187A	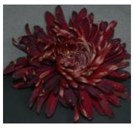
38	Andechixing	RHS53C	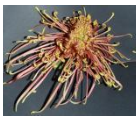
39	Bolanzhuangkuo	RHS49D	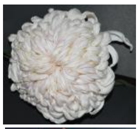
40	Gushuihonghe	RHS53A	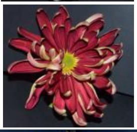
41	Musehanyan	RHS38D	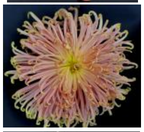
42	Dengxiawuniang	RHSN74C	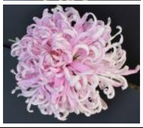

43	Mohe	RHS59A	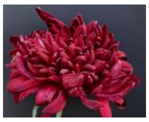
44	Jianliuhong	RHS70C	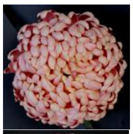
45	Zhangangudian	RHS64D	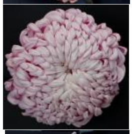
46	Fengliuxiaosa	RHS49D	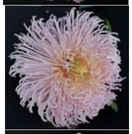
47	Jiangliuxiangfen	RHSN74C	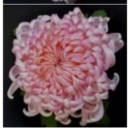
48	Caihuhonghuazhuang	RHS39B	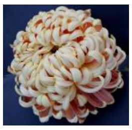
49	Huihezhanchi	RHSN74C	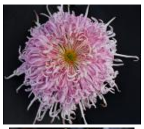
50	Shengguangdechao	RHS64D	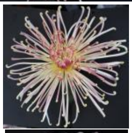
51	Guohuashengzhe	RHS64D	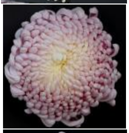
52	Quanxiangxianle	RHS56C	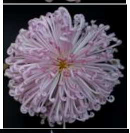
53	Yitaiyingzi	RHS49D	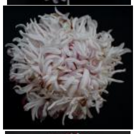
54	Panlongmeixiu	RHSN74C	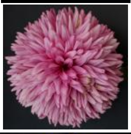
55	Quanxiangcanyangtian	RHS26D	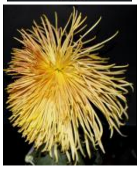
56	Panlongxinqiu	RHS39B	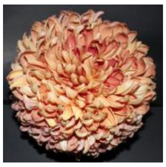

57	Quanxiangyuyin	RHSN155B	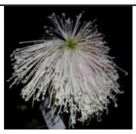
58	Guohuameishen	RHS65B	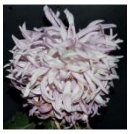
59	Pushuiliubing	RHS155D	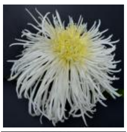
60	Guohuabaiyueshan	RHS155A	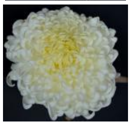
61	Qianshouguangyin	RHS155B	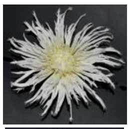
62	Taiyezhaoxue	RHSN999D	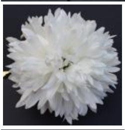
63	Panlongbaizhaoqiu	RHSN999D	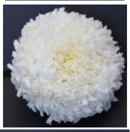
64	Tianshenglizhi	RHSN999D	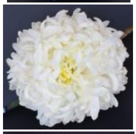
65	Qingfengbolv	RHS155A	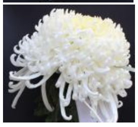
66	Xiangzhaochenguang	RHS157B	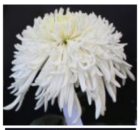
67	Bohexiang	RHSN999D	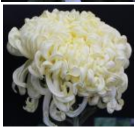
68	Shanwuyinshe	RHSN155D	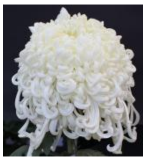
69	Xueyan	RHSN999D	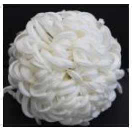
70	Baiyueshan	RHS155C	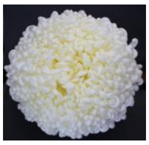

71	Guohuayinyueshan	RHSN999D	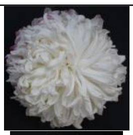
72	Changfengwanli	RHS155D	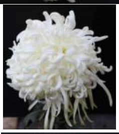
73	Yulupantao	RHSN155B	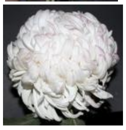
74	Lvyunqinghua	RHS155A	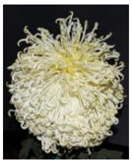
75	Qingbolengcui	RHS155C	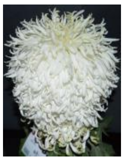
76	Baikuilong	RHSN999D	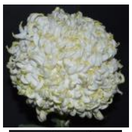
77	Panlongmulan	RHSN999D	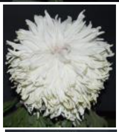
78	Panlongbiyu	RHSN999D	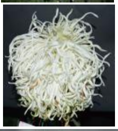
79	Ziyunfeiyue	RHSN79C	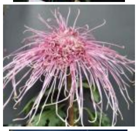
80	Qingjiandegeren	RHS72B	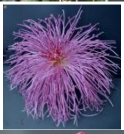
81	Guohuaqiangda	RHSN74C	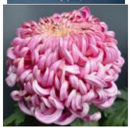
82	Jingyanruizi	RHSN79C	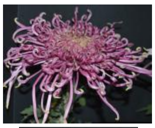
83	Panlongshenglian	RHS77B	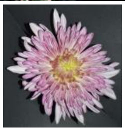
84	Yanzhipian	RHS71A	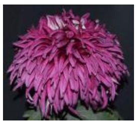

85	Luanshuiziyun	RHS72B	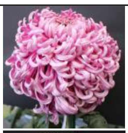
86	Jianliuxiangzi	RHSN74C	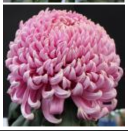
87	Yipinhong	RHSN79C	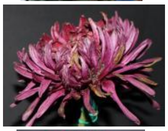
88	Zuiwoxiangyun	RHSN79C	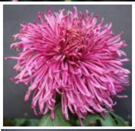
89	Xingyunyinglv	RHS145A	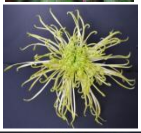
90	Gushuilvping	RHS145C	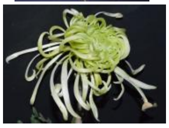
91	Fengpiaolvyi	RHS145C	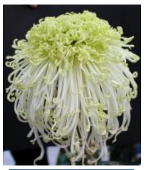
92	Baoxinglanxi	RHS45A	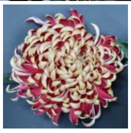
93	Dahongtuogui	RHS14A,45A	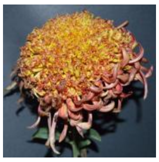
94	Lijin	RHS45A,6A	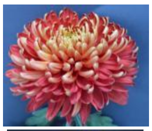
95	Taipingdehongye	RHS32A,6A	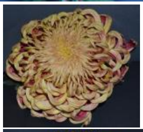
96	Fenghuangzhenyu	RHS45A,14A	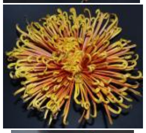
97	Moyu	RHS53A, N74C	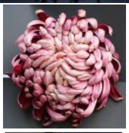
98	Jinpengdoucai	RHS23C,7D	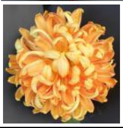

99	Huanshuimingzhu	RHS155C,4A	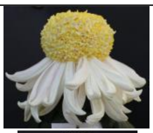
100	Guohuaqingwutai	RHS70C,4D	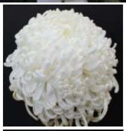
101	Xiangyunchunyu	RHS56A, N155B	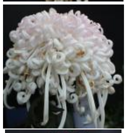
102	Qinghongaoshi	RHSN74C, N155B	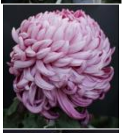
103	Yunluandiecui	RHS2C,155A	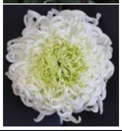
104	Tianma	RHS8C,4D	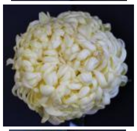
105	Guohuaxingran	RHS47A,7D	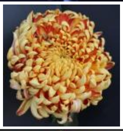
106	Wanshanhongbian	RHS53A,15D	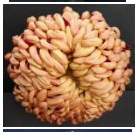
107	Panlongcangjing	RHS31D,15D	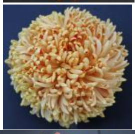
108	Fenglinwanxiu	RHS47A,31D	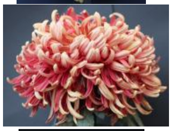
109	Quanxiangsiwo	RHS65B,155C	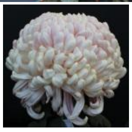
110	Ziyunzhuiyu	RHS71A, N155B	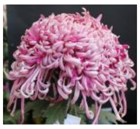
111	Shengguangbaochuan	RHS72B, N74C	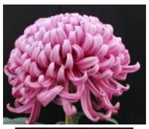
112	Tangyuqiushi	RHS72B, N155B	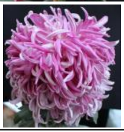

113	Cuifeng	RHSN77B,8C	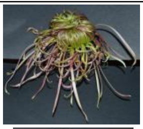
114	Caiyunzhuiyu	RHS72B, N155D	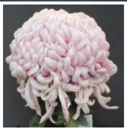
115	Caihumeiyu	RHSN74C	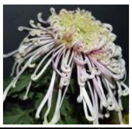
116	Hechengxinghuo	RHS22A	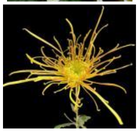
117	Quanxiangshuichang	RHS145C	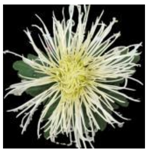

Supplementary Table 2 Transcriptome sequencing data.

CD	MCDL	ROI	MRLOI	NFNR	AFNRL	FLP
8,658,873	1754	450,789	2359	363,653	2162	80.67%

CD clean reads data, **MCDL** mean length of clean reads data, **ROI** read of insert, **MRLI** mean read length of insert, **MRQI** mean read quality of insert, **NFNR** number of full-length non-chimeric reads, **AFNRL** average full-length nonchimeric read length, **FLP** full-length percentage.

Supplementary Table 4 Functional annotation of the transcripts containing SSR markers.

SSR Marker Name	Unigene ID	Swissprot_Annotation
CHS-1	PB.21490.7	Chalcone synthase OS=Callistephus chinensis GN=CHS PE=2 SV=2
CHS-3	PB.4350.2	Chalcone synthase OS=Callistephus chinensis GN=CHS PE=2 SV=2
PSY-1a<br>PSY-1b	PB.20308.2	Bifunctional 15-cis-phytoene synthase, chromoplastic OS=Capsicum annuum GN=PSY1 PE=1 SV=1
LCYE-1	PB.32127.1	Lycopene epsilon cyclase, chloroplastic OS=Arabidopsis thaliana GN=LUT2 PE=1 SV=2
LCYE-4	PB.18776.1	Lycopene epsilon cyclase, chloroplastic OS=Solanum lycopersicum GN=CRTL-E-1 PE=2 SV=1
CHI	PB.15887.3	Chalcone--flavonone isomerase 1 OS=Chrysanthemum morifolium GN=CHI1 PE=2 SV=2
3MaT	PB.36973.1	Malonyl-coenzyme A:anthocyanin 3-O-glucoside-6"-O-malonyl transferase OS=Dahlia pinnata GN=3MAT PE=1 SV=1
